# The Mount Vernon Hospital Dispute

**Published:** 1913-07-19

**Authors:** 


### THE MOUNT VERNON HOSPITAL DISPUTE.

Some short time ago it will be remembered that a difference of opinion was manifested between the governing body and the medical staff of this institution as to the best method of reconstituting its system to meet the changed conditions caused by the Insurance Act, diminished subscriptions, and other new factors in the situation. In spite of the representations of the medical board, the committee of management decided to sell the Hampstead Hospital and to concentrate on the sanatorium at Northwood; and this step at the time we felt bound to approve as more likely to conduce to the future prosperity of the hospital than the policy desired by the medical staff. Now, however, a fresh dispute has arisen which has resulted in the resignation of a majority of the medical staff, who have not merely decided to sever their connection with it, but have also hinted pretty plainly at the possibility of legal proceedings for redress of their wrongs. They have, further, called in question the whole management of the institution. To begin with, it is to be noted that the document in which these views are set forth is not signed by all the staff. Dr. Squire, Dr. Lister, and Dr. Mackenzie, for example, are missing from the signatories, so presumably they do not feel any substantial share in the grievances of their colleagues. On the other hand, Sir H. Weber, Sir T. C. Allbutt, and Sir W. W. Cheyne—the consulting staff—have all signed, and evidently they feel that their colleagues of the active staff are being harshly treated.

The gist of the contention would seem to be that two of the staff officiating at Northwood have been curtly informed by the committee that their services are no longer required there; and this procedure certainly justifies the use of the term "summary dismissal," to which Mr. Charles Johnston, the chairman of the committee, objects as misleading. The latter's explanation is that owing to the impending sale of the Hampstead Hospital the committee thought well to place the beds at Northwood under the care of some of the senior members of the medical staff. The junior members would not have had long to wait for re-installation, as the Northwood Hospital is to be added to shortly and will provide beds for all the staff, both seniors and juniors alike.

To this scheme the dissentient doctors object, and it is easy to comprehend some of the reasons for their dissatisfaction. The manner in which the plans of the committee of management were intimated to them certainly appears to have been rude and tactless; and the doctors say it is not even in accordance with the by-laws of the hospital, a point upon which we do not here pronounce. Their allegations of mismanagement by the committee require investigation; and these, too, it is not for us to adjudicate. But with their contention that the committee cannot at their discretion select those members of the staff who shall be asked to do specified duties in the reorganised scheme of work we have no sympathy whatever. Probably the St. John's Hospital case, recently decided, has stiffened their backs; but we are wholly against the attitude—which we presume to be that of the resigning staff—that in such a process of selection the committee are acting *ultra vires*. Both sides of the disputation, it seems to us, have taken false steps which it will be difficult to retrace now. We trust, however, that some compromise acceptable to all parties may be possible without the resort to litigation.

